# Prevalence of Plantar Heel Pain Among School Teachers in Medina Region, Saudi Arabia: A Cross-Sectional Study

**DOI:** 10.7759/cureus.31821

**Published:** 2022-11-23

**Authors:** Yousef Alrashidi, Ehab F Alsaygh, Mohammed S Khoshhal, Obaid F Alsaedi, Baraa A Dwmlou, Hamza A Alandijani, Hussain R Aynusah, Mohammed S Aloufi, Hatim K Omar, Muhammad A Tobaiqi

**Affiliations:** 1 Department of Orthopedics, Taibah University, Medina, SAU; 2 College of Medicine, Taibah University, Medina, SAU

**Keywords:** plantar heel pain, musculoskeletal disorders, teachers, heel pain, foot pain

## Abstract

Background

Plantar heel pain (PHP) can be a common medical complaint among people with both sedentary and active lifestyles due to varied causes. It can affect the quality of life and result in significant disability. Despite many studies on PHP, few have focused on a specific population, such as school teachers. School teachers represent a significant proportion of the population of Medina, and addressing such a complaint and its possible relevant factors, which are most likely to be common among them due to their comparable job duties, will aid us in determining the relationships between personal characteristics, work-related factors, and PHP, as well as in formulating management plans. This study aims to identify the prevalence of PHP and its determinants among school teachers in the Medina region of Saudi Arabia.

Methodology

This cross-sectional study aims to identify the prevalence of PHP in school teachers. It was conducted in the Medina region of Saudi Arabia. A self-administered, online, validated questionnaire was created and used for data collection. Consent was taken from all participants before answering the questionnaire. Participation was voluntary, and all participants could withdraw from the study at any time. Data were kept confidential and only accessible by the primary investigator, co-investigators, and the statistician; hence, secondary and tertiary blinding was not done.

Results

Among those who reported PHP, the highest prevalence was among those who did not exercise regularly (94.7%), followed by middle-aged women (64.3%) and those with a high body mass index (44.5%), previous foot problems (43.2%), and chronic medical diseases (41.9%). PHP was less prevalent in male teachers, those with normal body mass index, and those who spent less time standing, had no previous foot problems, and exercised regularly. Most (88.1%) participants with PHP had other musculoskeletal pain, particularly in the lower back (62.6%) and knee (40.1%).

Conclusions

Teachers can be apprised about the importance of consuming a well-balanced diet and exercising regularly to maintain a healthy weight. We advocate educational programs as they can assist people to understand the need to obtain medical help when they are experiencing pain.

## Introduction

Plantar heel pain (PHP) is a frequent complaint among the general population, especially among middle-aged and elderly people. In the United States, almost 2 million people visit primary health clinics for PHP every year [1], and the condition affects one out of every 10 persons, accounting for approximately 11-15% of adult foot symptoms requiring medical treatment [2]. In Saudi Arabia, a study conducted among school teachers in Abha city revealed a prevalence of 17-24% of PHP [3]. Foot pain can negatively affect patients’ quality of life and lead to considerable disability. Although the prognosis of PHP is unclear, most symptoms seem to fade over time [4]. Despite numerous studies on PHP, few have examined specific populations, such as school teachers, whose jobs require frequent and prolonged standing. Because school teachers are considered a significant proportion of the population of Medina, addressing such an issue and its possible contributing factors, which are, to some extent, common among them due to their comparable job factors and duties, will aid us in determining the relationships between personal characteristics, work-related factors, and PHP, as well as in formulating management plans. In this study, the authors aimed to measure the prevalence of PHP and its determinants among school teachers in a single Saudi Arabian region.

One of the common causes of PHP is plantar fasciitis, typically described as a throbbing in the medial plantar heel, which worsens when taking the first steps in the morning, subsides temporarily on exercising and moving, and returns with greater intensity after extended weight-bearing. Other common causes include entrapment of the calcaneal, tibial, or Baxter’s nerve (the first branch of the lateral plantar nerve), which may lead to persistent pain and numbness on the dermatome of the target nerve, and stress fractures of the calcaneus, which usually accompany relevant trauma or stress-related activity and present as heel pain, point tenderness to palpation of the posterior calcaneus, and swelling at the fracture site. Fat pad atrophy of the heel is another cause of PHP which manifests as deep or bruise-like pain while standing, walking, or running, especially on a hard flat surface. Examination and palpation of the medial calcaneal tuberosity and along the plantar fascia typically cause pain [1]. Passive dorsiflexion of the foot and toes can also cause discomfort in certain people [5]. PHP may be referred in nature due to radiculopathy secondary to seronegative spondyloarthropathies. It includes level one radiculopathy of the sacrum, which is a numbness that runs down the back of the leg and into the outer or bottom of the foot [6]. Biomechanical characteristics such as obesity, limb length disparity, increased eversion at the subtalar joint, ankle joint range of motion, heel pad thickness, high arch height, and foot type such as flat foot are considered intrinsic risk factors for PHP. Extrinsic factors include prolonged work-related weight-bearing, increased exercise levels, and unsuitable shoes [7].

The patient’s medical history, including symptom onset and features of pain, can help the physician determine the exact cause of PHP. For example, Braxton nerve neuropathy causes acute radiating pain along Baxter’s nerve course, which worsens with physical activity (e.g., walking) and at night [8]. A patient with S1 radiculopathy will most likely have a history of long-term low back pain radiating down to the heel and a positive straight leg raise test [9]. Some etiologies require imaging studies in addition to history and physical examination to make a proper diagnosis. Weight-bearing X-ray, magnetic resonance imaging (MRI), and ultrasonography are the most commonly used modalities for diagnosis [10].

PHP remission has been observed in 60-80% of patients within 12-24 months of diagnosis, and the prognosis is considered favorable [11]. The most commonly recommended therapies are footwear modification, activity modification, taping, stretching exercises, anti-inflammatory agents, extracorporeal shock wave therapy, orthoses, and cortisone injections [12].

## Materials and methods

Study design

This cross-sectional study was conducted in the Medina region from January 2021 to the end of September 2021. Data were collected using the Google Forms online platform (www.docs.google.com/forms). A valid, structured, self-administered questionnaire was distributed online to the targeted population. The questionnaire, which was reviewed by a board-certified orthopedic surgeon and family physician for any conflicts or disagreements, comprised three sections covering demographic information, questions related to work and medical comorbidities, and PHP and how to analyze it.

Study participants

Of the 517 participants who were sent questionnaires, 52 were excluded either due to fulfilling some of the exclusion criteria, such as retired teachers or those in administrative positions, or due to inconsistent personal characteristics, such as incompatible weight and height measurements. Of the 465 participants included in this study, 94% and 6% worked in public schools and private schools in Medina, respectively. Male and female teachers in both public and private schools were included.

Data management and analysis

The retrieved data were tabulated and statistically analyzed using SPSS Statistics version 20 (IBM Corp., Armonk, NY, USA). The alpha level was set to 0.05, and all tests were two-sided. Multivariate logistic regression was used to assess possible risk factors associated with the presence of PHP.

Ethical considerations

Consent was taken from all participants before answering the questionnaire. Data were kept confidential and were only accessible by the primary investigator, co-investigators, and the statistician; hence, secondary and tertiary blinding was not done. Participation was voluntary, and all participants could withdraw from the study at any time. Ethical approval for this study was received from the Scientific Research Ethics Committee of the College of Medicine at Taibah University, Medina, Saudi Arabia (STU-20-018).

## Results

Of the 465 participants who responded to the questionnaire, 240 (52%) were women, 168 (36%) were obese (body mass index (BMI) ≥30 kg/m^2^), the mean age was 44 years (standard deviation (SD) = 6.8), 438 (94%) worked in a public school, and 129 (28%) had a previous foot problem. Overall, 36% of the respondents worked in elementary schools, 33% in middle schools, and 31% in high schools. The median weekly number of teaching hours was 16 (interquartile range = 8-20).

Chronic diseases (p = 0.054) were reported by 174 (37%) respondents, with diabetes mellitus (12%, p = 0.636) and hypertension (11%, p = 0.441) being the most common. Regarding exercise habits among respondents (p = <0.001), 13% reported that they never exercised, 43% reported that they exercised one time per week, 34% exercised two to three times a week, and 10% reported exercising more than three times a week. Regarding shoe type (p = 0.66), 35% of the respondents reported wearing sports shoes, 30% wore regular-type shoes, 23% wore sandals, 8% wore medical-type shoes, and 4% wore high heels. Table 1 summarizes the baseline characteristics of the study sample.

**Table 1:** Baseline characteristics of the studied sample. IQR: interquartile range

	Total N (%) or median (IQR)	No plantar heel pain (%) or median (IQR)	Plantar heel pain N (%) or median (IQR)	P-value
Number of cases	465	238 (51%)	227 (49%)	
Age (in years)	44 (40–48)	44 (39–49.25)	44 (40–48)	0.938
Male gender	225 (48%)	144 (60.5%)	81 (35.7%)	<0.001
Obesity (body mass index ≥30 kg/m^2^)	168 (36%)	67 (28.2%)	101 (44.5%)	<0.001
School type (public)	438 (94%)	222 (93.3%)	216 (95.2%)	0.387
Education stage				0.459
Elementary	166 (36%)	80 (33.6%)	86 (37.9%)	-
Middle	156 (33%)	79 (33.2%)	77 (33.9%)	-
High	143 (31%)	79 (33.2%)	64 (28.2%)	-
Exercise session				<0.001
Never	59 (13%)	24 (10.1%)	35 (15.4%)	-
Rarely (1 per week)	200 (43%)	88 (37%)	112 (49.3%)	-
Sometimes (2–3 per week)	160 (34%)	92 (38.7%)	68 (30%)	-
Always (>3 per week)	46 (10%)	34 (14.3%)	12 (5.3%)	-
Previous foot problem	129 (28%)	31 (13%)	98 (43.2%)	<0.001
Chronic disease	174 (37%)	79 (33.2%)	95 (41.9%)	0.054
Diabetes mellitus	56 (12%)	27 (11.3%)	29 (12.8%)	0.636
Hypertension	52 (11%)	24 (10.1%)	28 (12.3%)	0.441
Disc prolapse	41 (9%)	15 (6.3%)	26 (11.5%)	0.05
Gout	32 (7%)	13 (5.5%)	19 (8.4%)	0.216
Rheumatic disease	15 (3%)	3 (1.3%)	12 (5.3%)	0.014
Other	37 (8%)	15 (6.3%)	22 (9.7%)	0.177
Shoe type				0.66
Regular	140 (30%)	74 (31.1%)	66 (29.1%)	-
Sandals	108 (23%)	60 (25.2%)	48 (21.1%)	-
Sports	161 (35%)	78 (32.8%)	83 (36.6%)	-
Medical	37 (8%)	16 (6.7%)	21 (9.3%)	-
High heels	19 (4.1%)	10 (4.2%)	9 (4%)	-
Experience (years)	15 (10–21)	16 (10–22)	14 (10–20)	0.21
Teaching hours (per week)	16 (8–20)	16 (8–20)	16 (7–20)	0.809

Of the 465 respondents, 227 reported PHP. Of those, 34% reported that PHP did not affect their daily life, 53% that it sometimes affected their daily life, and 13% that PHP regularly affected their daily life. Most respondents (88%) with PHP reported having pain in other sites of the body, and 39% sought medical advice for PHP. Few respondents (10%) reported that PHP increased after shifting to online education after the coronavirus disease 2019 (COVID-19) pandemic. Table 2 summarizes the results.

**Table 2:** Pain intensity and other characteristics in respondents who reported plantar heel pain.

	N	%
Pain intensity		
Mild	70	30.8
Moderate	123	54.2
Severe	34	15
Foot affected		
Right	45	19.8
Left	57	25.1
Both	125	55.1
Pain affecting life activities		
No	76	33.5
Sometimes	121	53.3
Yes	30	13.2
Sought medical advice	89	39.2
Time of maximal pain		
Most of the time	38	16.7
After standing for a long time	138	60.8
After exercise	31	13.7
Early morning	53	23.3
After rest	52	22.9
Other times	8	3.5
Other pain sites	200	88.1
Low back pain	142	62.6
Knee	91	40.1
Neck	60	26.4
Shoulders	72	31.7
Other sites	8	3.5
Change in pain after online education		
Decreased	140	74.9
Same	29	15.5
Increased	18	9.6

To assess the possible risk factors associated with the presence of PHP, a multivariate sequential logistic regression was performed using the predictor variables shown in Table 3. The results showed that previous foot problems (odds ratio (OR) = 4.73; 95% confidence interval (CI) = 2.9-7.8; p < 0.001), working in public schools (OR = 3.40; 95% CI = 1.3-9.07; p = 0.014), being female (OR = 3.03; 95% CI = 1.97- 4.86; p < 0.001), and obesity (BMI ≥30; OR = 1.95; 95% CI = 1.25-3.02; p = 0.003) had statistically significant associations with PHP and could be considered risk factors for this condition.

**Table 3:** Multivariate sequential logistic regression on the presence of plantar heel pain. *: Compared to participants who never exercised. OR: odds ratio; CI: confidence interval

	Plantar heel pain (adjusted OR)	95% CI (adjusted OR)	P-value
Female gender	3.03	1.97–4.86	<0.001
Age >40 years	0.79	0.5–1.26	0.323
Chronic diseases	1.08	0.69–1.7	0.739
Obesity (body mass index ≥30 kg/m^2^)	1.95	1.25–3.02	0.003
Previous foot problems	4.73	2.9–7.8	<0.001
Public school	3.4	1.3–9.07	0.014
Weekly teaching hours	1.003	0.98–1.03	0.843
Exercise*	0.719	0.37–1.4	0.332
Rarely	0.518	0.264–1.01	0.056
Sometimes	0.351	0.14–0.88	0.026
Always	-	-	-

Being older than 40 (OR = 0.79; 95% CI = 0.5-1.26; p = 0.323), having a chronic disease (OR = 1.08; 95% CI = 0.69-1.7; p = 0.739), and number of weekly teaching hours (OR = 1.00; 95% CI = 0.98-1.03; p = 0.843) were not statistically significantly associated with PHP. Regarding exercise habits, participants who reported exercising more than three times a week had significantly lower odds of PHP compared to participants who never exercised (OR = 0.351; 95% CI = 0.14-0.88; p = 0.026).

## Discussion

The present findings indicate that PHP is a common issue among teachers aged 40-48 years. A high prevalence of PHP (49% of participants) in this study occurred among women over 40 years old. Of the 59 participants who never exercised, 35 had PHP compared to only 12 of the 46 participants who reported exercising at least three times per week (OR = 0.351; 95% CI = 0.14-0.88; p = 0.026).

Alqahtani administered the Posterior Heel Pain Health Survey to identify the prevalence and associated factors of foot pain among school teachers in Abha City, Saudi Arabia [3]. Among 1,439 teachers aged 24-60 years, 85.5% had foot pain, and those with foot pain were more likely to report pain in the low back, knee, neck, shoulder, and wrist. Most (79.4%) reported that foot pain affected their daily living, but only 17.7% sought medical advice. Similar to this study, Alqahtani also found a significant relationship between foot pain and activity level. Unlike the current study, Alqahtani’s study showed significant associations of PHP with long periods of standing and the number of teaching sessions per week. Alqahtani’s study also did not find significant associations of pain with female gender, obesity, working in public schools, and previous foot problems, as found in this study.

Tojo et al. identified foot and ankle pain prevalence among nurses at a Japanese university hospital using the Manchester Foot Pain and Disability Index [13]. Their questionnaire included a drawing to identify 26 areas of pain across the whole foot, including plantar, dorsal, and posterior aspects and the ankle. They also focused on psychosocial factors related to participants’ jobs by administering the Job Content Questionnaire to calculate a job strain index (psychological demands score divided by job control score). They assessed the degree of shoe comfort using a visual analog scale. Their estimated foot and ankle pain prevalence was 23% compared to 49% of participants in our study. In addition, only 4% of nurses reported that pain restricted their daily living compared to 17% who reported that it affected their work. The authors concluded that shoe comfort, individual factors (e.g., age and BMI), and psychosocial factors (e.g., high job strain and low job control) had significant relationships with foot and ankle pain. In contrast, the results of this study showed that pain was not significantly related to working hours or workload, which may be attributed to the same median of teaching hours reported by the two groups that may result in difficulty in concluding the effect of this variable. Further research is needed to compare factors influencing PHP in these job settings (teaching and nursing).

Thomas et al. surveyed the prevalence of PHP among 5,109 adults aged ≥50 years at one of four general practices in North Staffordshire, United Kingdom. The prevalence of PHP was 9.6% and slightly more prevalent among women, similar to our findings. PHP also was comparable across different age groups and significantly more prevalent in those who worked at repetitive-task occupations or were overweight, as well as those who reported wearing high-heeled footwear. They also assessed the mental aspects related to PHP and showed significant associations with mental impairment, anxiety, and depression [14].

McClinton et al. conducted a cross-sectional study to compare low back pain among individuals with and without PHP using the foot and ankle ability measure and Oswestry low back disability questionnaire [15]. Similar to this study, their results showed a higher prevalence of low back pain (74% vs. 37% of controls, OR = 5.2, p = 0.009) and low back disability (17% higher Oswestry score than controls, p < 0.001) among those with PHP. Neither age nor BMI was a statistically significant factor for individuals with PHP.

Some limitations of this study should be noted, such as a relatively small sample size. Moreover, due to the cross‐sectional study design, temporal aspects of these associations and causality could not be determined. No blinding was done in investigations, and any possible bias cannot be ruled out. The study did not identify the outcome of the medical treatment taken and it did not explore PHP management. In addition, variables such as types of shoes and modification of lifestyle during the COVID-19 pandemic were not discussed in detail. Nevertheless, the findings underscore the clinical implications of PHP associated with gender, weight, type of school, history of foot problems, and level of activity. Further research is needed to determine how these factors can cause or prevent PHP and to explore other factors that could be associated with PHP.

Preventive measures may help decrease the burden of this condition. To that end, future research should explore the effects of weight loss and exercise in preventing and treating PHP. These studies should also account for potential differences related to gender, considering the higher prevalence among women. Surprisingly, among those who reported wearing sports or medical shoes, rates of PHP were higher rather than lower, and more than 60% of individuals who reported wearing sandals and regular shoes were unaffected by PHP. This finding may be attributed to the lack of detail related to the use of each type, such as good comprehension of each type, duration, and type of activities done while wearing a certain shoe type. It would be interesting to investigate this finding further while adjusting other variables and providing questions to explore details about this variable. Finally, nine out of 10 participants reported having other pain sites, which is an interesting finding that needs further research to identify common factors associated with these complaints.

## Conclusions

The findings of this study suggest that people, especially obese women with a history of previous foot problems, engaged in professions that require prolonged standing or weight-bearing are more prone to experience PHP than other people in the population of Medina region. Therefore, additional research to further study the relationship of PHP to these factors and recommended management plans for possible causes of PHP should be conducted to improve the outcome of patients with PHP.

## Figures and Tables

**Figure 1:**
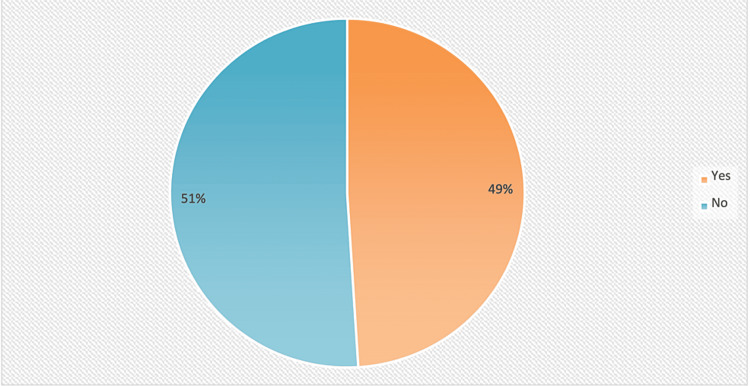
Plantar heel pain prevalence among teachers in the Medina region, Saudi Arabia.

**Figure 2:**
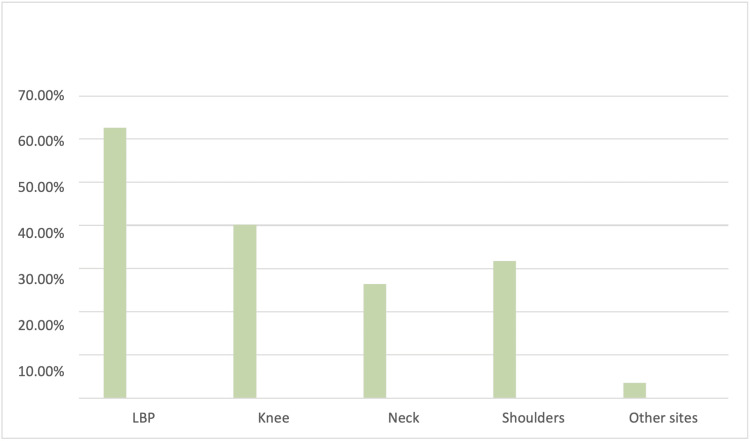
Pain in other sites among teachers with plantar heel pain in Medina region, Saudi Arabia. LBP: lower back pain

**Figure 3:**
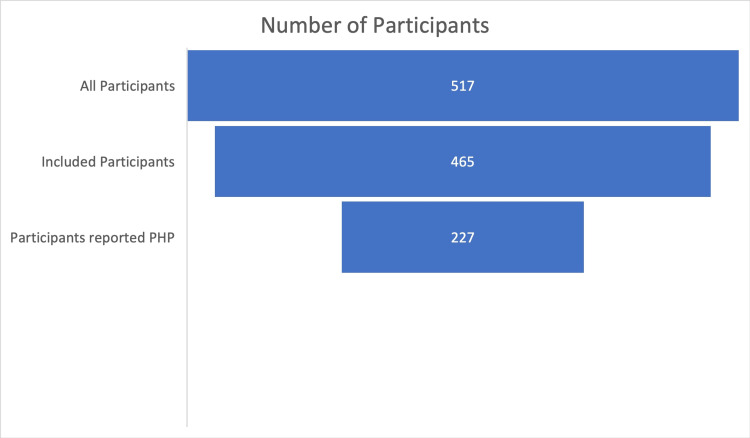
Number of participants in the study.
